# Annexin‐1 is an oncogene in glioblastoma and causes tumour immune escape through the indirect upregulation of interleukin‐8

**DOI:** 10.1111/jcmm.17458

**Published:** 2022-06-29

**Authors:** Rui Chen, Chengqi Chen, Na Han, Wenjing Guo, Hui Deng, Yali Wang, Yanpeng Ding, Mengxian Zhang

**Affiliations:** ^1^ Department of Oncology, Tongji Hospital, Tongji Medical College Huazhong University of Science and Technology Wuhan China; ^2^ Department of Hepatobiliary Surgery The First Affiliated Hospital of Xi'an Jiaotong University Xi'an China; ^3^ Department of Oncology, The Second Clinical Medical College Yangtze University Jingzhou China; ^4^ Department of Oncology, Zhongnan Hospital Wuhan university Wuhan China

**Keywords:** annexin‐1, dendritic cell, glioblastoma, interleukin‐8, NF‐κB

## Abstract

Annexin‐1 (ANXA1) is widely reported to be deregulated in various cancers and is involved in tumorigenesis. However, its effects on glioblastoma (GBM) remain unclear. Using immunohistochemistry with tissue microarrays, we showed that ANXA1 was overexpressed in GBM, positively correlated with higher World Health Organization (WHO) grades of glioma, and negatively associated with poor survival. To further explore its role and the underlying molecular mechanism in GBM, we constructed ANXA1shRNA U87 and U251 cell lines for further experiments. ANXA1 downregulation suppressed GBM cell proliferation, migration, and invasion and enhanced their radiosensitivity. Furthermore, we determined that ANXA1 was involved in dendritic cell (DC) maturation in patients with GBM and that DC infiltration was inversely proportional to GBM prognosis. Considering that previous reports have shown that Interleukin‐8 (IL‐8) is associated with DC migration and maturation and is correlated with NF‐κB transcriptional regulation, we examined IL‐8 and p65 subunit expressions and p65 phosphorylation levels in GBM cells under an ANXA1 knockdown. These results suggest that ANXA1 significantly promotes IL‐8 production and p65 phosphorylation levels. We inferred that ANXA1 is a potential biomarker and a candidate therapeutic target for GBM treatment and may mediate tumour immune escape through NF‐kB (p65) activation and IL‐8 upregulation.

## 1 INTRODUCTION

Glioblastoma (GBM) is the most common and deadliest primary tumour in the central nervous system, accounting for 50% of diffuse gliomas and having strong invasion ability, rapid growth and inexorable recurrence.^1^, ^2^

During the past decades, despite the emergence of more accurate molecular classifications and advancements in treatment methods for GBM, patients still have very short median survival times of 12–18 months.^3^ Therefore, more studies are needed to identify specific molecules and explore the potential regulatory mechanisms underlying GBM to provide new therapeutic targets.

Annexin A1 (ANXA1), a calcium‐dependent phospholipid‐binding protein, was initially discovered to be a glucocorticoid‐regulated anti‐inflammatory protein.^4^ Further in‐depth studies revealed that ANXA1 is deregulated in a large number of cancers and is involved in multiple cancer processes, including cell proliferation, invasion, metastasis and radiosensitivity.^5^ However, studies have found that ANXA1 expression is different depending on tumour type and that ANXA1 acts as either an oncogene or a suppressor gene.^6^ Previous studies have reported that, even in the same breast cancer, the expression and role of ANXA1 vary in different subtypes. Okano et al. demonstrated that ANXA1 expression increased in triple‐negative breast cancer and was associated with its aggressive features.^7^ In contrast, another study suggested that ANXA1 downregulated in progressed human breast cancer and acted as a suppressor of both epithelial‐mesenchymal transition and metastasis in murine and human cells and tumours.^8^ Additionally, ANXA1 localization in the nucleus, cytoplasm and membrane could also be a clinical factor determining ANXA1 roles and regulatory mechanisms in cancer.^9^ In gliomas, ANXA1 expression significantly increased in poorly differentiated human primary gliomas compared with normal brain tissue or lower‐grade tumours, indicating that ANXA1 is associated with the development and progression of malignant glioma.^10^ Thus, it is conceivable that ANXA1 acts as an oncogene in GBM and is correlated with poor patient outcomes. Nevertheless, the comprehensive effects of ANXA1 on GBM biology remain unclear. Accordingly, further research should focus on the regulatory mechanisms of ANXA1 in GBM.

Interleukin‐8 (IL‐8), a CXC chemokine and also known as CXCL8, was initially described as an inflammatory innate immune response mediator and subsequently shown to be a potent angiogenic factor leading to tumour progression and metastasis in various human cancers.^11^ Binding with its highly related CXCR1 and CXCR2 receptors, IL‐8 participates in tumour immune responses, angiogenesis and cellular proliferation.^12^, ^13^ Several studies have reported that IL‐8 is produced by a variety of tumours and always leads to more invasive cancer phenotypes, including that of GBM.^14^ Generally, IL‐8 gene expression is regulated by the transcriptional activation of nuclear factor‐kB (NF‐κB), which is suggested to be mainly associated with the binding between the minimal promoter region of IL‐8 and the p65 or cRel homodimers of NF‐κB.^15^, ^16^ Recently, it was found that IL‐8 could affect dendritic cell (DC) maturation by retaining DCs in tumours and blocking the migration capacity of DCs from initial inflammation sites to draining lymph nodes, affecting its antigen‐presenting function in tumours.^17^, ^18^, ^19^ Based on the literature and previous reports, we hypothesized that NF‐κB (p65) hyperactivation mediated by ANXA1 is partly responsible for IL‐8 overexpression, which inhibits DC maturation in GBM.

This study aimed to elucidate the role of ANXA1 in GBM biology, including GBM cell proliferation, invasion, migration and apoptosis, and to provide new insights into the mechanism of ANXA1‐mediated tumorigenicity by which ANXA1 increases IL‐8 expression through NF‐κB (p65) activation, thereby affecting DC functions and finally leading to immune escape in GBM.

## 2 MATERIAL AND METHODS

### 2.1 Immunohistochemical (IHC) staining and evaluation

Two tissue microarrays (TMAs), including HBraG125PG02 and HBraG171Su01, consisting of 270 samples of different glioma grades, and three normal brain tissue samples were purchased from Shanghai Outdo Biotech Co., Ltd. for IHC analysis. After removal of samples decolourised during immunohistochemical staining for ANXA1 and CD1a, the HBraG171Su01 microarray, which included 149 glioma samples, had CD1a and PD‐L1 staining results, but lacked information on tumour invasion and tumour size, and the HBraG125PG02 microarray, which included 121 tumour samples and 3 normal control samples, had information on PD‐L1, EGFR, and ki67 expression, but lacked survival information.

All paraffin‐embedded tissues were dewaxed in xylene and washed with gradient ethanol concentrations. After retrieving antigens by heating tissues in a microwave oven, slides were blocked with 5% bovine serum albumin (BSA) in PBS for 30 min at room temperature. Subsequently, slides were incubated with primary rabbit monoclonal anti‐ANXA1 (Cell Signaling Technology) at 4 °C overnight and secondary goat anti‐rabbit (Dako Cytomation, Glostrup, Denmark) for 45 min. Finally, tissue sections were stained with haematoxylin, and the ANXA1 expression level in tissues was evaluated by the H‐score. The final staining score for each tissue depended on both the staining intensity and percentage of positive cells. The former was scored as follows: 0, negative staining; 1, weak staining; 2, moderate staining; and 3, strong staining; and the latter was scored as follows: 0, no staining; 1, <25%; 2, 25%‐50%; 3, 50%‐75%; and 4, >75% of cells are stained. The multiplied result of the two scores represented protein levels, and tumour tissues with H‐scores greater than the median of all scored tumour tissues were classified as having a high molecular expression.

### 2.2 Bioinformatics analysis

To verify the expression and prognostic role of ANXA1 in patients with GBM, several online bioinformatics analysis tools were used. We first used GEPIA2 (http://gepia2.cancer‐pku.cn/#index) and the oncomine database (https://www.oncomine.org/resource/main.html) to analyse the differential expression of ANXA1 based on The Cancer Genome Atlas (TCGA) GBM dataset, with a P threshold value of 1E‐4, a fold change of 2, and a gene rank of the top 10%. CGGA (Chinese Glioma Genome Atlas, http://www.cgga.org.cn/index.jsp ) was used to determine the distribution of ANXA1 in patients with gliomas. TIMER2.0 (http://timer.comp‐genomics.org/) and TISIDB (http://cis.hku.hk/TISIDB/index.php) were used to explore the correlation between ANXA1 and immune infiltrates. Additionally, we also used TIMER2.0 to show the effect of differential ANXA1 levels and immune infiltrates on the prognosis of patients with GBM. Gene set enrichment analysis (GSEA, version 4.1.0) was used to predict the potential roles of ANXA1 in the immune system. A *p*‐value <0.05 was considered statistically significant.

### 2.3 Cell culture and lentivirus transduction

The human glioblastoma cell lines U251 and U87 used in this study were purchased from the National Infrastructure of Cell Line Resource (Beijing, China). All cell lines were grown in DMEM (Hyclone, USA) supplemented with 10% foetal bovine serum (FBS) (Gibco, USA) and incubated at 37 °C in a humidified atmosphere of 5% CO_2_.

Lentiviral GV248 vectors expressing ANXA1 shRNA and scramble non‐target shRNA were established by Genechem Co (Shanghai, China). The target sequence for human ANXA1 shRNA was selected as 5' ‐ GCCTCACAGCTATCGTGAA ‐ 3', and the negative control sequence was 5' ‐TTCTCCGAACGTGTCACGT ‐ 3'. The lentivirus infection was manipulated referring to instructions. Finally, according to the verification of the ANXA1 expression by Western blotting and a real‐time (RT)‐polymerase chain reaction (PCR) test, U251 and U87 cells with ANXA1 downregulation were harvested for later research.

### 2.4 Cell proliferation and colony formation assay

To measure GBM cell proliferation, we performed a CCK‐8 assay. First, 2000 cells/well were plated in a 96‐well plate for 24 h before counting. Then, 10 μL of a Cell Counting Kit‐8 reagent (Boster, China) was added to each well. After an incubation period of 2 h, the absorbance of each well was measured at a wavelength of 450 nm using a spectrophotometer. This process was repeated on days 2, 3, and 4. Finally, a growth curve was drawn using Graphpad Prism 5.0. For the colony formation assay, 500 cells from each well were evenly seeded into 6‐well plates and then incubated at 37 °C in a 5% CO_2_ atmosphere for 2 weeks. Cell clones were harvested for 1% crystal violet staining and counting when visible clones appeared in a petri dish. A cluster with more than 50 cells was considered a positive clone.

### 2.5 Cell migration and invasion assay

Cell migration was determined using a scratch wound healing assay. Cells were first seeded as monolayers in 6‐well plates with perpendicular markings on the bottom surface as reference points for imaging. After reaching 90%–100% confluence, cells were scraped with a 200 μl pipette tip. After additionally growing for 18 and 36 h, cells were stained with crystal violet in methanol for 30 min. Subsequently, wound healing ability was measured by counting the distance between the two sides of the scratch using Image‐Pro Plus software version 6.0. The distance at 0 h was considered the control value. Images were taken at 0, 12 and 24 h after wounding under an inverted microscope.

To compare the invasion ability of the U251 and U87 cells that were stably transduced with ANXA1 shRNA and NC shRNA, the transwell invasion assay was performed by seeding 200μl (1 × 10^6^ cell/mL) of cells into 24‐well Transwell chambers with a pore size of 8 μm precoated with Matrigel (BD Biosciences). A medium containing 5% FBS was added to the lower chamber as a chemoattractant. After incubation at 37 °C for 24 h, cells on the lower side were washed with PBS, fixed with 4% paraformaldehyde and stained with 0.1% crystal violet. Subsequently, cells were counted in five randomly selected fields from each membrane under an inverted microscope.

### 2.6 Western blotting

Total protein from GBM cells was extracted using a radioimmunoprecipitation assay lysis buffer. After determining total protein concentration with a BCA Protein Assay Kit (Beyotime Institute of Biotechnology), equal amounts of extracts were separated by sodium dodecyl sulphate‐polyacrylamide gel electrophoresis in a 10% resolving gel at 100 V for approximately 2 h (loading volume of 20 μg), and then transferred to polyvinylidene difluoride membranes (Biosharp, China). Next, the membrane was blocked using Tris‐buffered saline with Tween (TBST) containing 5% milk and incubated with primary ANXA1 (Abcam), p65 (Proteintech, China) and p‐p65 (Abclonal, China) antibodies overnight at 4 °C. After extensive washing, secondary antibodies conjugated with horseradish peroxidase (Biosharp, China) were added for 1 h at room temperature. Finally, immunoreactive protein bands were detected using enhanced chemiluminescence reagents and quantified using ImageJ software. Glyceraldehyde 3‐phosphate dehydrogenase (GAPDH, Santa Cruz Biotechnology) and β‐actin (Abcam) were used as an endogenous loading control to calculate relative protein levels.

### 2.7 Quantitative RT‐PCR (qRT‐PCR) analysis

Total RNA was extracted from U251 and U87 cells using TRIzol (Clontech), and complementary DNA was synthesized using a reverse transcription reagent kit (Thermo scientific). Next, real‐time PCR amplification was performed using SYBR Green PCR Master Mix (TaKaRa, Japan). The primers used were as follows: ANXA1, 5'‐GCGGTGAGCCCCTATCCTA/TGATGGTTGCTTCATCCAC‐3'; IL‐8, 5’‐TTTTGCCAAGGAGTGCTAAAGA/AACCCTCTGCACCCAGTTTTC‐3′; GAPDH, 5’‐ACAACTTTGGTATCGTGGAAGG/GCCATCACGCCACAGTTTC‐3′. GAPDH was used as an endogenous control. Relative gene expression levels were calculated using the comparative cycle threshold method, in which the relative expression was calculated using the ΔΔCq method.

### 2.8 Enzyme‐linked immunosorbent assay (ELISA)

IL‐8 ELISA was performed using the Human IL‐8 ELISA kit from BioLegend following the manufacturer's instructions. Briefly, cells (1 × 10^6^ cells/well) transfected with ANXA1 shRNA and NC shRNA were plated in a 6‐well plate. After incubation at 37 °C for 72 h, an equal volume of cell culture supernatants was collected. Furthermore, IL‐8 concentrations in culture supernatants were measured by absorbance at a 450‐nm wavelength and determined by comparing their optical density with the standard curve.

### 2.9 Statistical analyses

SPSS version 22.0 (IBM Co., Armonk, NY, USA) was used for all data analyses. The two‐tailed paired Student’s *t*‐test or chi‐square test was used to compare differences between the cancer and control groups. Associations between ANXA1 status and clinicopathological characteristics from different samples were assessed using Fisher's exact test or the x^2^ test. Univariate and multivariate analyses were conducted using a Cox logistic regression analysis to identify independent variables. Overall survival (OS) and progress‐free survival (PFS) curves were plotted using the Kaplan–Meier method and compared using the log‐rank test. All quoted *p*‐values are two‐tailed, and *p*‐values <0.05 were considered statistically significant. All experiments were conducted at least three times.

## 3 RESULTS

### 3.1 High ANXA1 expression in patients with GBM and the association of ANXA1 with clinicopathological factors

**FIGURE 1:**
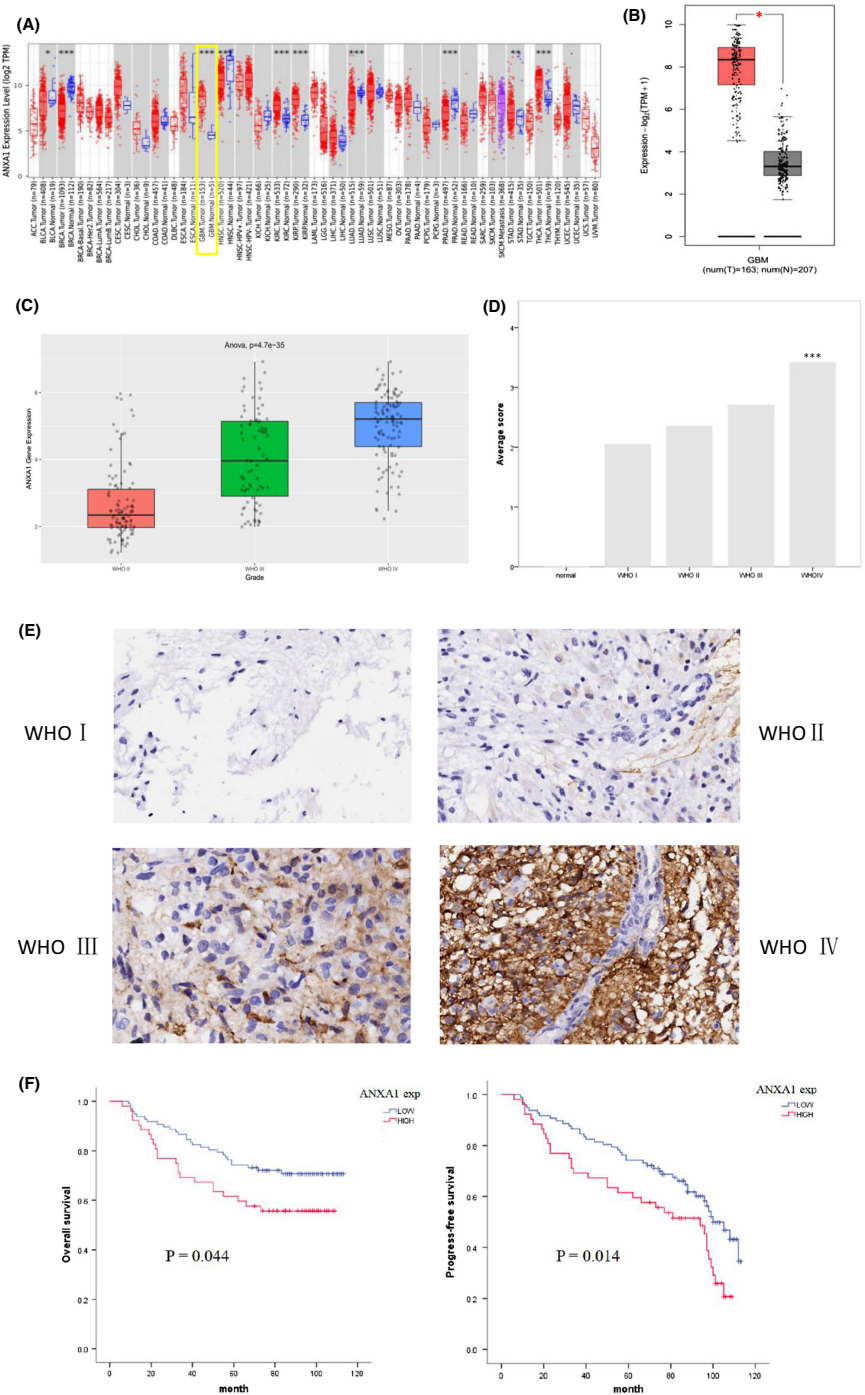
ANXA1 expression and survival analysis in GBM. (A), The differential expression analysis of ANXA1 between tumour and adjacent normal tissues in pan‐cancer. (B), Comparative analysis of ANXA1 gene expression in GBM group compared with in normal group by GEPIA2. (C), ANXA1 gene expression of different WHO grade glioma based on CGGA database. (D), Average H‐score of ANXA1 expression of different WHO grade glioma tissues and normal brain tissues. (E), Representative ANXA1 IHC staining of glioma tissues and corresponding adjacent normal tissues. Positive ANXA1 staining was found in cytoplasmic tumour cells. (F), Kaplan–Meier survival curves show progress‐free survival (PFS) and overall survival (OS) for the ANXA1 low expression group and high expression group based on immunohistochemical analysis (*p* < 0.05). The log‐rank test shows that GBM patients with high ANXA1 expression have lower OS (left) and PFS (right) than those with low expression of ANXA1. *p < 0.05; **p < 0.01; ***p < 0.001

ANXA1 expression level distributions plot, in a pan‐cancer analysis by TIMER, showed that ANXA1 was obviously elevated in a variety of tumours, including GBM, compared with ANXA1 expression in normal groups (Figure 1A). Next, we validated this result using GEPIA, a more comprehensive tool integrating gene expression profile data from TCGA and GTEx projects. ANXA1 overexpression in GBM has also been observed (Figure 1B). Furthermore, to evaluate ANXA1 expression in gliomas of different grades, analysis using the CGGA database, a user‐friendly web application for data storage and analysis to explore brain tumour datasets over 2,000 samples from Chinese cohorts, showed that a higher ANXA1 expression was significantly associated with higher histologic grades (Figure 1C). It is worthy to note that, based on the immunohistochemical staining of two tissue microarrays, results verified again that average ANXA1 immunostaining scores were significantly higher in GBM than in low‐grade gliomas (LGG) or normal tissues (Figure 1D). Additionally, morphological results of the immunohistochemistry assay showed that ANXA1 upregulates with an increase in tumour grade (Figure 1E). Therefore, these results strongly indicate an association between ANXA1 levels and advanced grades of glioma. To further assess potential prognostic roles of ANXA1 in GBM, a Kaplan–Meier survival analysis of the clinical information of tissue microarrays showed that ANXA1 expression was inversely correlated with OS and PFS (Figure 1F). Consistently, the KM curve from the Gene_Outcome module of TIMER 2.0 indicated that ANXA1 was a poor GBM prognostic factor (Figure S1B). Similar to the Outcome module, Gene_Surv, a Cox proportional hazard model in TIMER 2.0, and the survival analysis in CGGA showed the significance of ANXA1 expression as an optional clinical predictive factor (Table S1 and Figure S1A).

Correlations between clinicopathological features and ANXA1 expression based on information from two tissue microarray samples were explored using the x^2^ test; these correlations are summarized in Table 1. There were significant differences between low and high ANXA1 expression based on age (*p* = 0.002), tumour location (*p* = 0.001), recurrence (*p* = 0.048), WHO grade (*p* = 0.005), CD1a (*p* = 0.015) and PD‐L1 expression (*p* = 0.000); however, other parameters were not correlated with this difference. Moreover, high expression of ANXA1 were associated with high WHO grade, as well as high CD1a and PD‐L1 expression in gliomas. We investigated the association between ANXA1 and PD‐L1 and EGFR expression using Pearson correlation and linear regression analyses. Results showed that ANXA1 expression was positively correlated with PD‐L1 expression (*r* = 0.332, *p* < 0.01), but not with EGFR expression (*r* = 0.152, *p* < 0.052, Figure S1C). A univariate Cox regression model revealed that age (>40 years), recurrence, a high WHO grade, and a high expression of CD1a, ANXA1 and EGFR were negatively correlated with the OS of patients with GBM. Additionally, multivariate analysis confirmed that age (>40 years), and a high WHO grade and ki67 index could be independent OS prognostic factors in glioma patients, with hazard ratios of 0.241, 0.149 and 2.282, respectively (Table 2). Together, these results suggest that high ANXA1 expressions are correlated with glioma aggressiveness.

**TABLE 1:** Correlation of ANXA1 expression with clinicopathological factors

Factors	*n*	ANXA1‐low	ANXA1‐high	*p*‐value
Age(y)				
≤40	111	84	27	0.002***
>40	159	91	68	
Gender				
Male	180	115	65	0.652
Female	90	60	30	
Tumour location				
Frontal	93	73	20	0.001**
Parietal	18	15	3	
Temporal	89	49	40	
Occipital	18	8	10	
Others	52	30	22	
Tumour size (cm)				
≤5	85	53	32	0.451
>5	36	25	11	
Tumour invasion				
Yes	32	20	12	0.787
No	89	58	31	
Recurrence				
Yes	81	47	34	0.048*
No	68	50	18	
WHO grade				
I	24	19	5	0.005**
II	100	73	27	
III	73	47	26	
GBM	73	36	37	
CD1a^a^				
Low	107	76	31	0.015*
High	42	21	21	
PD‐L1^a^				
Low	176	133	43	0.000***
High	94	42	52	
EGFR^a^				
Low	88	60	28	0.343
High	61	37	24	
Ki67^a^				
Low	79	49	30	0.403
High	70	48	22	

*p < 0.05, **p < 0.01, ***p < 0.001.

^a^ Data were expressed as median.

**TABLE 2:** Univariate and multivariate cox proportional hazards analysis of OS

Variable	n	Univariate analysis			Multivariate analysis		
		HR	95% CI	*p*‐value	HR	95% CI	*p*‐value
Age(y)							
≤40	64						
>40	85	0.260	0.130–0.519	0.000***	0.241	0.105–0.553	0.001***
Gender							
Female	50						
Male	99	0.612	0.326–1.149	0.126	0.789	0.385–1.616	0.517
Recurrence							
No	71						
Yes	78	0.010	0.001–0.102	0.000***	0.000	0.000–2.106E+60	0.878
WHO grade							
I	19						
II	66	0.000	0.000–1.335E+243	0.956	0.053	0.000–3.025E+134	0.985
III	45	0.019	0.008–0.050	0.000***	0.025	0.008–0.077	0.000***
GBM	19	0.145	0.074–0.284	0.000***	0.149	0.066–0.335	0.000***
ANXA1							
Low	97						
High	52	0.572	0.329–0.993	0.047*	1.925	0.855–4.334	0.114
CD1a							
Low	107						
High	42	0.414	0.237–0.721	0.002**	0.804	0.407–1.587	0.529
PD‐L1							
Low	105						
High	44	0.903	0.500–1.632	0.736	1.709	0.767–3.809	1.190
EGFR							
Low	88						
High	61	0.541	0.312–0.937	0.028*	1.008	0.538–1.891	0.980
Ki67							
Low	79						
High	70	0.951	0.549–1.647	0.859	2.282	1.138–4.576	0.020*

Abbreviation: HR, hazard ratio.

*p < 0.05, **p < 0.01, ***p < 0.001.

### 3.2 ANXA1 knockdown suppressed GBM cell proliferation, migration and invasion

**FIGURE 2:**
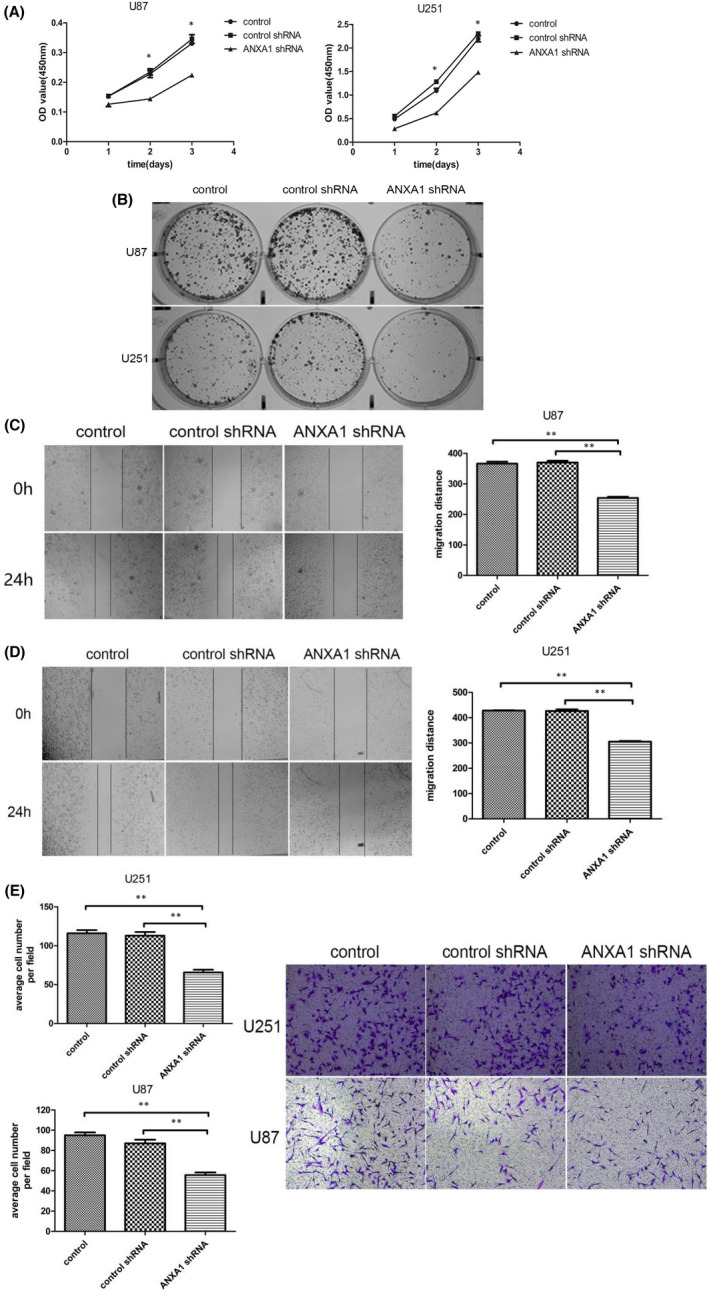
Role of ANXA1 in the proliferation, cell clones, migration and invasion of GBM cell lines. (A), CCK‐8 assays show that ANXA1 downregulation decreased cell proliferation in ANXA1shRNA U87 and U251 cell lines cells. (*p < 0.05). (B), Representative images of clone formation on plastic plates with U87 and U251cell lines expressing ANXA1shRNA or empty vector or control cells, which indicates significantly decreased the number of colonies in the ANXA1shRNA cell lines compared with another two control groups. (C) and (D), Wound healing assays show that ANXA1 knockdown significantly reduced the cell migration ability of U87 and U251 cells with the representative images on the left and the quantitative analysis on the right (**p < 0.01). (E), Transwell invasion of ANXA1shRNA GBM cells is significantly reduced compared with control cells. All experiments were repeated thrice. **p < 0.05

As mentioned before, the potential roles of ANXA1 as an oncogene in patients with GBM were revealed based on clinical parameters. To subsequently identify the biological functions of ANXA1 in patients with GBM, we selected two GBM cell lines (U251 and U87) with high expressions of ANXA1 for ANXA1 knockdown experiments according to qRT‐PCR and Western blot assays (Figure S2A and B). After stable ANXA1 shRNA transfections, U251 and U87 cells were subjected to qRT‐PCR and Western blot again; this verified that ANXA1 expressions were significantly reduced in both cell lines compared with untransfected parental cell, indicating an effective lentivirus‐delivered shRNA sequence (Figure S2C and D). Thereafter, we first performed CCK8 and soft agar colony formation assays to investigate the effect of ANXA1 on the viability and clonability of GBM cells. Silencing ANXA1 delayed U251 and U87 proliferation, and the number of cell clones was lower in the ANXA1 knockdown group than in the control group (Figure 2A and B). These results suggest that silencing ANXA1 suppresses GBM cell growth. Moreover, to clarify the role of ANXA1 downregulation in the migration of GBM cells, wound healing and transwell assays were performed. We observed that after ANXA1‐shRNA transfections for 24 h, cell migration was reduced significantly compared to that in the control group (Figure 2C and D). As for the invasion assay, the number of cells that passed through the membrane into the lower side of the chamber in the ANXA1 knockdown groups was significantly lower than that in the control group (Figure 2E). Together, these results confirmed that cell migration and invasion were significantly inhibited by ANXA1 downregulation in GBM cells, indicating the importance of ANXA1 in GBM progression.

### 3.3 Correlation between ANXA1 gene expression and immune infiltrates in patients with GBM

**FIGURE 3:**
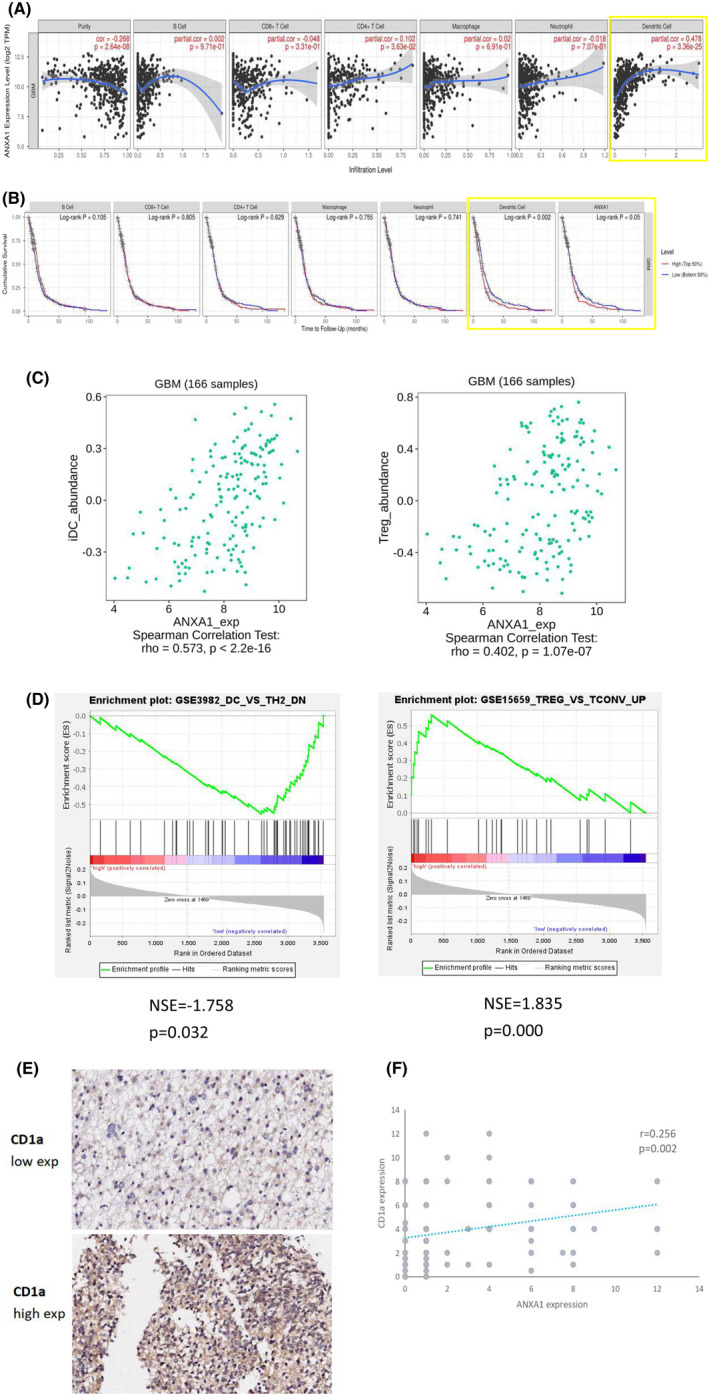
The correlation between ANXA1 and tumour immune inflitration in patients with GBM. (A), The association between the expression of ANXA1 in GBM and the abundance of six immune cell types (B cells, CD4+ T cells, CD8+ T cells, neutrophil, macrophage and myeloid dendritic cells) using the tool of TIMER. The yellow box represents the correlation between ANXA1 and myeloid dendritic cells (cor = 0.478, *p* < 0.001). (B), The prognostic value of ANXA1 (*p* = 0.05) and immature dendritic cells (*p* = 0.002) in GBM based on TIMER. (C), Correlation analysis of the infiltration level of immature dendritic cell (iDC) or Treg cell in GBM with the expression of ANXA1. The left shows ANXA1 and iDC with the correlation coefficient of 0.573 and the right represents ANXA1 and Treg cell, with the correlation coefficient of 0.402. (D), GSEA analysis indicates that DC related gene set (left) enriched in ANXA1 downregulation group (NES = −1.758, *p* = 0.032) and Treg related gene set (right) enriched in ANXA1 high expression group (NES = −1.835, *p* = 0.000). (E), Representative CD1a high and low IHC score of glioma tissues. Positive CD1a staining was found in cytoplasmic tumour cells. (F), Scatter plots of the expression correlation analysis of ANXA1 and CD1a based on IHC staining scores (*r* = 0.256, *p* < 0.05). Tumour tissues with H‐scores greater than the median of all scored tumour tissues were classified as high expression

Accumulating years of studies have shown the anti‐inflammatory activities of ANXA1 in several cancers. However, as no ANXA1 research to our knowledge included GBM, its immune function on GBM is still unclear. Therefore, we tried to determine whether ANXA1 is associated with the tumour‐immune system in patients with GBM. Using the TISIDB database to determine which types of TILs might be regulated by ANXA1, we found that ANXA1 might affect the immune regulation of various tumours, including GBM (Figure S1D). Concurrently, it was found that the infiltration level of immature DCs (iDCs) in patients with GBM was the most closely related to ANXA1 among other parameters, with a correlation coefficient of 0.573, followed by Treg cells, with a correlation coefficient of 0.402 (Figure 3C). Subsequently, the TIMER tool was used to estimate the association between the expression of ANXA1 in GBM and the abundance of six immune cell types, including B, CD4+ T, CD8+ T cells, neutrophils, macrophages and myeloid DCs. Similarly, results showed that only myeloid DCs were closely related to ANXA1 expression (Figure 3A, cor = 0.478, *p* < 0.001). Moreover, similar to the effect of ANXA1 on GBM prognosis (*p* = 0.05), the higher the infiltration level of iDCs, the worse the prognosis (Figure 3B, *p* = 0.002). To explore biological functions associated with GBM immunity, which may be related to ANXA1, an ANXA1 GSEA analysis was performed. According to the median ANXA1 expression, all GBM transcriptional data in the TCGA database were first divided into high and low expression groups. With a nominal threshold *p*‐value of <0.01, we obtained only one gene set of “TREG_VS_TCONV_UP” enriched in the ANXA1 high expression group (NES = ‐1.835, *p* = 0.000). However, 10^6^ gene sets, in which was the “GSE3982_DC_VS_TH2_DN” gene set, were significantly enriched in GBM samples in the ANXA1 low expression group at a nominal *p*‐value < 0.05 (NES = ‐1.758, *p* = 0.032) (Figure 3D). To verify the correlation of ANXA1 with iDC and Treg, molecular markers of them (CD1a and Foxp3, respectively) were mapped and quantified using the tissue microarray HBraG171Su01. Immunohistochemical staining revealed that Foxp3 stained negatively in the cytoplasm, whereas CD1a stained in the cytoplasm as did ANXA1, and CD1a expression increased with ANXA1 (Figure 3E and F). The results obtained by these two methods were similar, indicating that ANXA1 expressions were positively correlated with the infiltration degree of Treg and iDCs, but negatively correlated with the number of mature DCs.

### 3.4 ANXA1 knockdown in GBM cells decreased NF‐κB activation and IL‐8 expression

**FIGURE 4:**
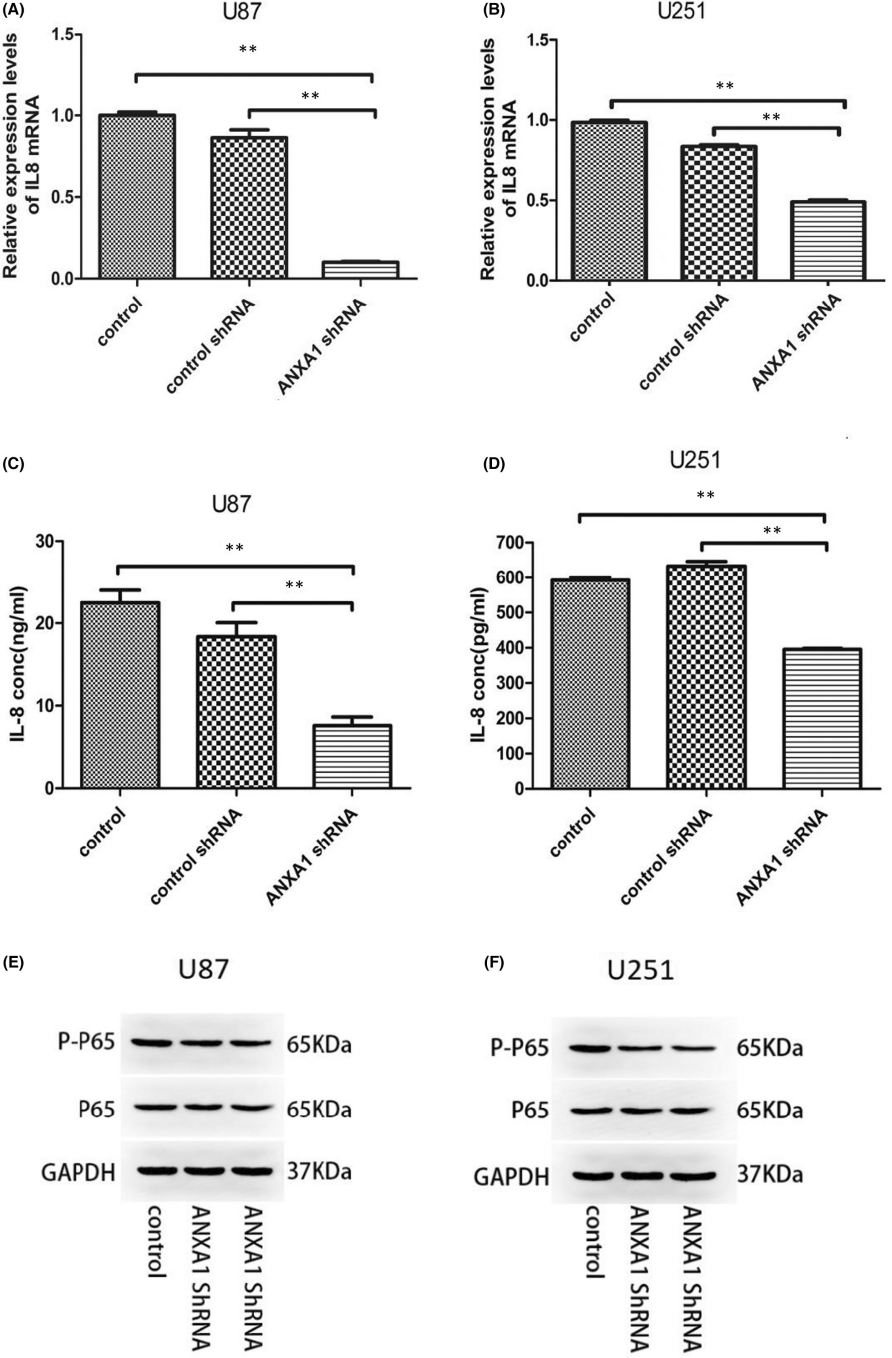
The regulation of IL‐8 and NF‐kB (p65) by ANXA1 in U87 and U251 cell lines. (A) and (B), Quantitative real‐time PCR analysis of IL‐8 mRNA expression in ANXA1shRNA U87 and U251 cells compared with controls (**p < 0.01). (C) and (D), IL‐8 secretion into the culture medium by cells expressing ANXA1shRNA or empty vector assessed by an enzyme‐linked immunosorbent assay (**p < 0.01). (E) and (F), The expression of ANXA1, p65 and phosphorylation p65 (p‐p65) in U87 and U251 cells transduced with lentivirus was examined by Western blot. GAPDH was an internal control. Control shRNA: GBM cells transduced with empty vector lentivirus; ANXA1 shRNA: GBM cells transduced with lentiviral GV248 vector expressing ANXA1 shRNA

The above evidence suggests that ANXA1 might influence DC maturation, thus mediating GBM immune escape. To investigate the mechanism by which ANXA1 regulates tumour immunity, we analysed the relationship between ANXA1 and immune molecules. In view of reports that IL‐8 may contribute to iDC maturation, we experimentally studied changes in IL‐8 expression before and after ANXA1 knockdown. As shown in Figure 4A and B, specific shRNA‐induced ANXA1 knockdown led to a marked decrease in IL‐8 mRNA expression in both U87 and U251 GBM cells compared with control cells. To further test whether IL‐8 concentrations in the medium underwent the same ANXA1 regulation, we performed ELISA using U87 and U251 cells. As expected, we found that levels of IL‐8 in GBM cells significantly decreased upon ANXA1 knockdown (Figure 4C and D). Thus, we suggest that there is a positive correlation between ANXA1 expression and IL‐8 in GBM cells. Additionally, we detected the p65 phosphorylation level to confirm NF‐κB activation in GBM cell lines upon ANXA1 knockdown through a Western blot. As shown in Figure 4E, with an ANXA1 knockdown, the p65 phosphorylation level decreased without changes in p65 expression. To confirm this result, we performed the same experiments using U251 cells and obtained similar results (Figure 4F). Therefore, we speculated that ANXA1 functions may be related to p65 phosphorylation as well as the activity of NF‐κB; such functions regulate IL‐8 expression.

## 4 DISCUSSION

Our current study confirms that ANXA1 is overexpressed in human GBM at both mRNA and protein levels and is correlated with high glioma grade and poor outcome. Furthermore, we not only examined the biological role of ANXA1 in GBM cell proliferation, migration, and invasion under ANXA1 knockdown, but we were also the first, to our knowledge, to elucidate the possible mechanism that ANXA1 affects NF‐κB transcriptional activity, and thus, IL‐8 expression to mediate GBM immune escape.

Previous studies have shown that ANXA1 is either upregulated in tumours such as gastric cancer,^17^, ^18^, ^19^, ^20^ pancreatic carcinoma^21^ and lung cancer^22^ and correlated with poor prognosis or downregulated in tumours such as head and neck squamous cell carcinoma^23^ and nasopharyngeal carcinoma.^24^ Typically, studies have focused on the effects of ANXA1 binding to the FPR receptor. For example, ANXA1 was shown to be involved in tumour microenvironment possibly by increasing M1 macrophages, and to control tumour growth and metastasis via its receptor FPR2 in breast cancer.^25^ Yang Y et al. found that ANXA1, as an FPR1 agonist, was more highly expressed in poorly differentiated human primary gliomas compared with lower‐grade tumours, and accounted for stimulating GBM cell growth and invasion.^10^ Consistently, our IHC data and cell experiment results both determins that ANXA1 may function as an oncogene in GBM. Moreover, high ANXA1 expression in GBM tissues is positively correlated with a higher WHO grade, recurrence rate and PD‐L1 expression, and is associated with poor OS and PFS. Our further functional ANXA1 data suggested that ANXA1 promoted GBM cell proliferation, migration and invasion; these functions are consistent with ANXA1 functions in many other cancers. Therefore, ANXA1 might be a promising target for new molecular therapy against GBM and attracted us to this exploration.

As an anti‐inflammatory protein, ANXA1 has always been associated with tumour immunity. The interaction between ANXA1 and FPR1 was suggested to influence intratumoral DC maturation and T cell‐mediated anticancer immune responses, thus abolishing the efficacy of anthracycline‐based chemotherapy in cancer.^26^ Baracco et al. showed that ANXA1‐deficent cancer cells exhibit a defect in the exposure of calreticulin (CALR), facilitating the phagocytic uptake of dead‐cell antigens by DC.^27^ In present study, we firstly evaluated the association of ANXA1 expression within the GBM microenvironment. According to TIMER2.0 and TISIDB databases, the results showed that ANXA1 was closely related to the infiltration degrees of iDCs and Treg cells. Our functional enrichment analysis demonstrated a corresponding finding. Notely, ANXA1 concentrations were proportional to iDC infiltration but inversely proportional to mature DC infiltration, suggesting that ANXA1 is an important mediator of mature DC migration. Most importantly, the correlation between ANXA1 and CD1a, a marker of iDC, was also confirmed by localization and quantitative analysis performed by immunohistochemical staining. Moreover, a high level of iDCs predicted poor GBM prognosis.

Considering the role of IL‐8 in DC maturation, our study verified ANXA1 influence on IL‐8 expression to determine the precise regulatory mechanism of ANXA1 underlying the tumour‐immune system in GBM. The results of the qRT‐PCR test followed by ELISA in our study later demonstrated that, compared with controls, ANXA1 depletion suppressed the production of IL‐8 in U251 and U87 cells. Therefore, there might be crosstalk between ANXA1 and IL‐8. Previous studies have reported that NF‐κB is a major transcriptional regulator capable of modulating IL‐8 expression in many types of cancer, including GBM.^28^, ^29^, ^30^, ^31^ In previous studies, ANXA1 has been reported to both activate and inhibit NF‐κB activity. For example, ANXA1 overexpression in the cytoplasm was shown to constitutively activate NF‐κB through the interaction with the IKK complex in breast cancer cells^32^, ^33^ and in NSCLC.^34^ However, another research suggested that ANXA1 associated with NF‐kappaB and suppressed its transcriptional activity by preventing NF‐kappaB binding to DNA in colon and pancreatic cancers.^35^ This echoes its role as either an oncogene‐promoting or oncogene‐suppressing gene in different tumours. Likewise, our data indicated that after silencing ANXA1 in U251 and U87 cells, the p65 phosphorylation level, which needs to be high for NF‐κB activation, was significantly decreased. Hence, we hypothesized that ANXA1 could increase IL‐8 levels in patients with GBM through aberrant NF‐κB activation, subsequently affecting DC maturation; this consequently impairs the antigen presentation function of DCs and suppresses immunity in patients with GBM. Subsequent Western blotting experiments confirmed this possibility.

Although our study is the first to report the possible relationship between ANXA1 and GBM immune escape, the specific mechanism by ANXA1 suppresses immunity still needs to be further studied. Therefore, this study has some limitations. First, there are only bioinformatic results regarding the relationship between ANXA1 and immune cells, especially DC infiltration, in patients with GBM. Additional experimental evidence is required to confirm this hypothesis. Second, it is not sufficient to prove the ANXA1‐NF‐kB‐IL‐8 axis based on the presently available experimental data. The exact mechanism by which ANXA1 affects NF‐κB transcriptional activity and the specific promoter region in which NF‐κB binds to IL‐8 require further investigation. Finally, in vivo studies are needed to further understand the biological role of ANXA1 in GBM. Collectively, our findings suggest that ANXA1 expression increases in patients with GBM and confers a poor patient prognosis. ANXA1 knockdowns inhibit GBM cell proliferation, migration and invasion and promote the apoptosis rate of U251 cells under radiotherapy, implying its function as a potential therapy for GBM treatment. Simultaneously, our systematic investigation identified a possible underlying mechanism by which tumour immune evasion is regulated by ANXA1 through activating the NF‐kB/IL‐8/DC pathway.

## AUTHOR CONTRIBUTIONS

**Mengxian Zhang:** Conceptualization (lead); funding acquisition (supporting); project administration (lead); resources (lead); supervision (lead); validation (lead); writing – review and editing (lead). **Rui Chen:** Data curation (equal); formal analysis (equal); investigation (equal); methodology (equal); resources (equal); software (lead); visualization (lead); writing – original draft (lead); writing – review and editing (equal). **Chengqi Chen:** Conceptualization (equal); data curation (equal); formal analysis (equal); methodology (equal); resources (supporting); supervision (equal); validation (equal); visualization (supporting); writing – review and editing (equal). **Na Han:** Conceptualization (equal); funding acquisition (supporting); investigation (equal); project administration (supporting); validation (supporting); writing – review and editing (supporting). **WenJing Guo:** Formal analysis (supporting); methodology (supporting); supervision (supporting); validation (supporting). **Hui Deng:** Data curation (supporting); formal analysis (supporting); software (supporting); validation (supporting). **Yali Wang:** Formal analysis (supporting); investigation (supporting); supervision (supporting); validation (supporting). **Yanpeng Ding:** Formal analysis (supporting); software (supporting); validation (supporting).

## CONFLICT OF INTEREST

No conflict of interest exists in the submission of this manuscript.

## Supporting information


Appendix S1

## Data Availability

All data generated or analysed during this study are included in this article.
