# Supplementary material for: Annexin‐1 is an oncogene in glioblastoma and causes tumour immune escape through the indirect upregulation of interleukin‐8
Source: J Cell Mol Med. 2022 Jun 29;26(15):4343–56. doi: 10.1111/jcmm.17458 (PMC9344830; doi:10.1111/jcmm.17458)
Supplement: Supplementary file 1 — Appendix S1 [file JCMM-26-4343-s001.pdf]

Table S1 Survival difference in GBM - Cox Proportional Hazard Model

ANXA1 in GBM (n=153):

Model: Surv(OS, EVENT) ~ `ANXA1` + Race + Gender

149 patients with 119 dying (4 missing obs.)

	coef	HR	se(coef)	95%CI_l	95%CI_u	z	p	signif
ANXA1	0.164	1.178	0.076	1.015	1.366	2.163	0.031	*
RaceBlack	0.671	1.957	0.724	0.473	8.089	0.927	0.354	
RaceWhite	0.095	1.099	0.602	0.338	3.580	0.157	0.875	
Gendermale	-0.064	0.938	0.197	0.638	1.381	-0.323	0.747	

Rsquare = 0.047 (max possible = 9.98e-01)

Likelihood ratio test p = 1.27e-01

Wald test p = 1.29e-01

Score (logrank) test p = 1.24e-01

Kaplan-Meier Curve Parameters:

Split Expression Percentage of Patients: 0-50(%)

Survival Time Between: 0-200(Month)

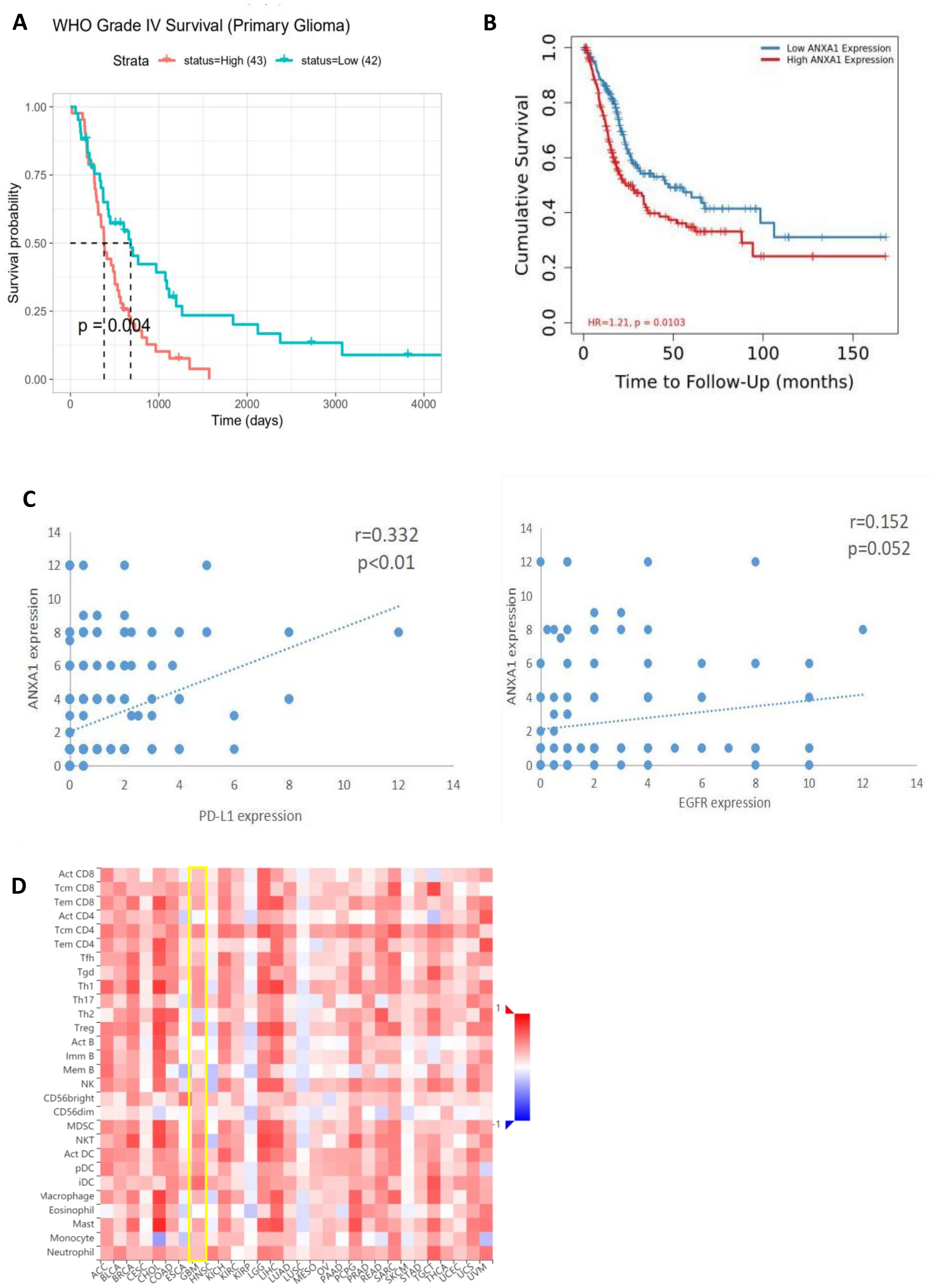
A
WHO Grade IV Survival (Primary Glioma)
Strata
status=High (43)
status=Low (42)
Survival probability
Time (days)
p = 0.004
B
Cumulative Survival
Time to Follow-Up (months)
Low ANXA1 Expression
High ANXA1 Expression
HR=1.21, p = 0.0103
C
r=0.332
p<0.01
ANXA1 expression
PD-L1 expression
r=0.152
p=0.052
ANXA1 expression
EGFR expression
D
Act CD8
Tcm CD8
Tem CD8
Act CD4
Tcm CD4
Tem CD4
Tfh
Tgd
Th1
Th17
Th2
Treg
Act B
Imm B
Mem B
NK
CD56bright
CD56dim
MDSC
NKT
Act DC
pDC
iDC
Macrophage
Eosinophil
Mast
Monocyte
Neutrophil
ACC BLCA BRCA CESC CHOL COAD ESCA GBM HNSC KICH KIRC KIRP LGG LIHC LUAD LUSC MESO OV PAAD PCPG PRAD READ SARC SKCM STAD TGCT THCA UCEC UCS UVM

FigureS1 A and B, Kaplan-Meier survival curves based on CGGA (A, $p = 0.004$) and TIMER 2.0 (B, $p=0.0103$, $HR=1.21$) show that primary GBM patients with low ANXA1 expression have better prognosis. C, Scatter plots of the expression correlation analysis of ANXA1 and PD-L1 (left, $r=0.332$, $p < 0.01$) or EGFR (right, $r=0.152$, $p = 0.052$) based on IHC staining scores. Tumor tissues with H-scores greater than the median of all scored tumor tissues were classified as high expression. D, The correlation investigation between the abundance of tumor-infiltrating lymphocytes (TILs) and ANXA1 expression through TISIDB database finds that ANXA1 might be involved in the immune regulation of varieties of tumors including GBM.

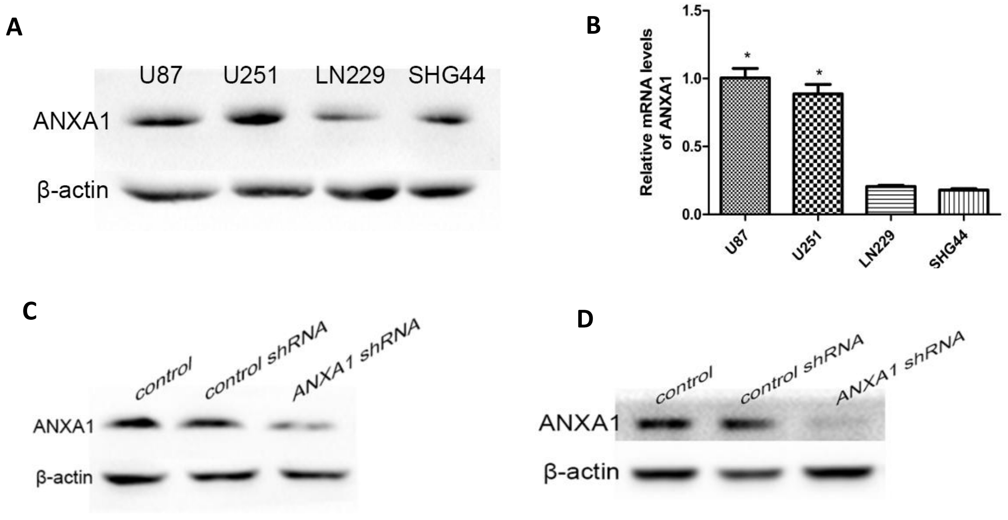

FigureS2 A and B, The expression of ANXA1 in four types GBM cells was determined by Western blotting (A) and qRT-PCR (B). β -actin was an internal control. C and D, The expression of ANXA1 in U87 and U251 cells transduced with lentivirus was examined by Western blot.
